# Risks of parasitic helminth disease outbreaks and influence of physico-chemical factors in flood prone areas of Yaoundé, Cameroon

**DOI:** 10.1016/j.parepi.2024.e00404

**Published:** 2024-12-16

**Authors:** Noura Efietngab Atembeh, Jean Patrick Molu, Jeanne Valerie Emvoutou Maboulou, Boris Fominyam, Rodrigue Nanfack Dongmo, Gabriel Bertrand Zambo, Emilie Tchinda Tiecheu, Jeannette Tombi, Lucia Nkengazong, Serge Hubert Zebaze Togouet, Jean Louis Essame Oyono

**Affiliations:** a Centre for Research on Health and Priority Pathologies, Institute of Medical Research and Medicinal Plants Studies, PO box 13033 Yaoundé, Cameroon; b Laboratory of Parasitology and Ecology, Faculty of Science, University of Yaoundé I, PO box 812 Yaoundé, Cameroon; c Laboratory of Hydrobiology and Environment, Faculty of Science, University of Yaoundé I, PO box 812 Yaoundé, Cameroon; d University of Yaoundé I, PO box 812 Yaoundé, Cameroon

**Keywords:** Helminthiases, Physicochemical factors, Flood areas, Yaoundé, Cameroon

## Abstract

**Background:**

Urbanization coupled with poverty has promoted the exploitation of risk zones like flood-prone areas in the city of Yaoundé. The overcrowding and poor hygiene observed in these areas are responsible for the unsmiling variations in environmental cleanliness, exploitation of river water for domestic purposes thus putting them at risk for parasitic disease transmissions. This study was conducted in order to assess the risks of human helminthiases outbreaks in relation to water physico-chemical factors in the city of Yaoundé.

**Method:**

Water samples were collected monthly from January to June 2022 in 12 stations from 4 rivers (Biyeme, Ebogo, Olezoa and Tongolo) situated in flood plains in the town of Yaoundé. Intestinal parasite resistant forms were identified using the basic sedimentation technique, while water parameters were assessed using standard procedures.

**Results:**

Results indicated that rivers in flood-prone areas are highly contaminated with a 75 % prevalence rate. In all, resistant forms of nine parasite species were recorded with the genus *Toxocara* being the most diversified with two species. *Strongyloides stercoralis* was the most prevalent (33.3 %) and most abundant (1269) species followed by *Toxocara canis* (26.4 %), *Ascaris lumbricoides* (25 %), *Toxocara leonina* (20.8 %), Hookworm (15.3 %), *Trichostrongylus* sp. (11.1 %), *Diphyllobothrium latum* (8.3 %), *Trichuris trichiura* (2.8 %) and *Schistosoma intercalatum* (1.4 %). The least abundant species was *Trichuris trichiura* (33). Higher densities of parasite resistant forms were observed during the dry season. River Tongolo was the most contaminated with pathogenic forms. Water pH, orthophosphate, organic matter and alkalinity revealed negative corelations with the occurrence of *Diphyllobotrium latum* (*r* = −0.375, −0.253, −462, −0.448 respectively) while organic matter and pH showed positive correlations with the occurrence of *Strongyloides stercoralis* (*r* = 0.378) and *Trichostrongylus* sp. (*r* = 0.238) respectively.

**Conclusion:**

Flood areas constitute a potential risk zone for the maintenance and spread of human helminthiases. Maintaining proper personal cleanliness, environmental sanitation through the building of functional and accessible faecal disposal facilities and drainages and abstaining from using the rivers as waste dumps are essential in this area to prevent parasitic helminth disease outbreaks.

## 1 Introduction

High rates of urbanization pose a significant issue, especially for African cities where poverty is concentrated and has resulted in unchecked urban growth and exploitation of protected regions like flood-prone zones (Bloch et al., 2012; Ahiablame and Shakya, 2016). It is often accompanied by an artificialization of urban rivers, which increases the risk of water overflows hence flooding (Douglas, 2017). Flood is defined as the inundation of a normally dry area caused by rising water in an existing waterway, such as a river, stream, or drainage ditch. Apart from the destruction of properties and human lives, flood may lead to severe damages in the environment (damage of biotopes, water and soil) (Zeleňáková et al., 2017). The damage of the water environment is explained by the changes in its physicochemical factors (for example temperature, organic matter, conductivity, suspended solids just to name a few) and may influence the survival, transmission, and spread of infectious diseases (Okaka and Odhiambo, 2018) among which are human helminthiases. Ajeagah et al. (Ajeagah et al., 2016) revealed that suspended solids, oxygen, pH and organic matter could positively impact the development of parasite resistant forms as they provide them with nutrients necessary for their survival (Ajeagah et al., 2016). Thus, an increase in the values of these parameters in the environment will lead to an increase in the amount of viable pathogenic forms hence increasing the probability of them infesting their intermediate and definitive hosts. Whilst Mbouombouo et al. (Mbouombouo et al., 2019), explained that high concentrations of mineral elements in the environment could increase their inactivation and destruction due to their high capacity to penetrate helminths' membrane (Mbouombouo et al., 2019). This environmental instability can be explained by the fact that during floods, water and sewage infrastructures are damaged extensively leading to the discharge of a considerable amount of untreated industrial, human and animal wastes into water bodies and soil, which causes the helminths' eggs and larvae to infest or contaminate these areas. As a result, raises the likelihood of contact with faeces laden with geohelminth eggs and exposes individuals to the possible risk of infections which is known to be acquired via the oral faecal route (WHO, 2023).

The population of Yaoundé has increased over the last 10 years as compared to most cameroonian towns, particularly in the metropolitan core. This population boom has been driven by factors such as natural rise and internal and external migration. When the town was founded in the 1890s, it had only 300 native people. Today, with 2.3 million residents (BUCREP, 2010) and growing exponentially at a rate of 4.1 % annually the population will double every 8 to 9 years (Tiafack and Mbon, 2017). This growth has led to anarchic urbanization that is exacerbated by poverty; homes are being built in areas that are prone to flooding and beside rivers, which, when combined with unsanitary conditions, may contaminate the water supply. Furthermore, due to the absence or poor availability of safe water, residents of some of these town areas have been forced to use river water for their domestic needs (drinking, swimming, laundry, cleaning cars, irrigating crops, etc.), putting them at risk for parasitic helminth infections. Owing to their great prevalence, global dispersion, and negative effects on an individual's nutritional state and immune system, parasitic helminths pose a significant threat to public health. Although children are the most susceptible individuals to these diseases, adults of all ages are also afflicted (WHO, 2023). The faecal-oral route and skin penetration, which are linked to sanitation and hygiene conditions, are the two ways that these parasites are transmitted.

Although studies have shown the presence of protozoans and bacteria in surface water in the city of Yaoundé (Ajeagah et al., 2016; Ajeagah et al., 2010; Ajeagah et al., 2019; Ngong et al., 2019; Ewoti et al., 2022; Asi et al., 2022), reports on the influence of physicochemical factors on the transmission dynamics of helminth eggs and larvae peculiar to flood zones remain few or poorly defined (Ajeagah and Fotseu Kouam, 2019). The purpose of this study was to assess the relationship between the physico-chemical factors of four rivers (Biyeme, Olezoa, Tongolo and Ebogo) and the risk of human helminthiases outbreaks, from flood zones in the town of Yaoundé. More specifically, to determine the presence of helminth eggs and larvae with time and assess the impact of physico-chemical parameters on parasite distribution in these waters.

## 2 Materials and methods

### 2.1 Description of study sites

**Fig. 1:**
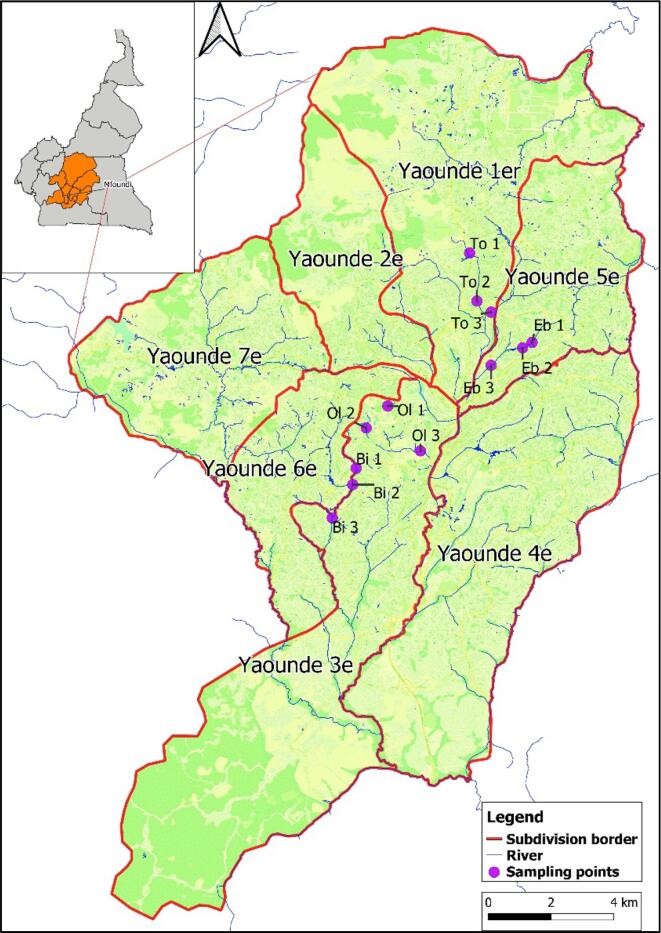
Geographical location of sampling sites (Source: the map was taken from https://data.humdata.org/ and modified using QGIS software version 3.16.0 Hannover).

This study was carried out in the Mfoundi Division of the Centre Region of Cameroon. With about 4.2 million people living there as of 2015, it has a total surface area of 69,000 km^2^ and houses the city of Yaoundé, the political capital of Cameroon. Yaoundé is situated in the south of the Centre region between latitude 3°30′ and 3°58’ North and between longitude 11°20′ and 11°40′ East and at an average altitude of 750 m (Suchel, 2024). It has a subequatorial climate with two dry seasons (a long dry season from mid-November to mid-March and short one from mid-June to mid-August) alternating with two wet seasons from September to mid-November (long rainy season) and mid-March to May (short rainy season). With an average rainfall of 1000–2000 mm per year, and an average temperature of 24 °C. It is characterised by its hydromorphic and brown or lateritic soil types (Suchel, 2024). Four (04) watersheds of the Mfoundi river were selected based on criteria such as the presence of a possible source of pollution, human settlement, accessibility and lowlands prone to floods (Table 1). For each watershed, 3 sampling stations were identified (upstream, middle and downstream) and named as follows: Biyeme (Bi 1, Bi 2, Bi 3), Tongolo (To 1, To 2, To 3), Olezoa (Ol 1, Ol 2, Ol 3) and Ebogo (Eb 1, Eb 2, Eb 3) (Fig. 1).

**Table 1:** Characteristics of sampling stations.

River	Stations	Geographic coordinates		Source of pollution	Human activities
		Latitude	Longitude		
Biyeme	Bi 1	3.84291	11.49198	Presence of a domestic waste dump	Commercial activities
	Bi 2	3.83821	11.491	Presence of piped latrines and a domestic waste dump	Car wash
	Bi 3	3.82866	11.48514	Open defecation and domestic pollution	Car wash
Olezoa	Ol 1	3.860755	11.50108	Presence of piped latrines and domestic waste are dumped into the river	Vegetable farming and irrigation
	Ol 2	3.85449	11.49486	Presence of piped latrines and domestic waste are dumped into the river	Commercial activities
	Ol 3	3.84791	11.51045	Presence of piped latrines and a domestic waste dump	Commercial activities
Ebogo	Eb 1	3.87905	11.54249	Presence of piped latrines and domestic waste are dumped into the river	Commercial activities
	Eb 2	3.87748	11.53974	Presence of piped latrines and domestic wastes are dumped into the river	Commercial activities
	Eb 3	3.872463	11.53072	Presence of piped latrines and domestic wastes are dumped into the river	Commercial activities
Tongolo	To 1	3.9047	11.52455	Presence of latrines, domestic animals and domestic wastes are dumped into the river	Commercial activities
	To 2	3.89092	11.52664	Presence of latrines, domestic animals and domestic wastes are dumped into the river	Commercial activities
	To 3	3.88773	11.53072	Presence of latrines, domestic animals and domestic wastes are dumped into the river	Commercial activities

Bi1 = biyeme 1; Bi2 = biyeme 2; Bi3 = biyeme 3; Eb1 = Ebogo 1; Eb2 = Ebogo 2; Eb3 = Ebogo 3; Ol1 = Olezoa 1; Ol2 = Olezoa 2; Ol3 = Olezoa 3; To1 = Tongolo 1; To2 = Tongolo 2; To3 = Tongolo 3.

### 2.2 Sample collection

A monthly sampling campaign was carried out on these watersheds for a period of six months (from January to June 2022). At each sampling river, 6 samples (3 for both physico-chemical and biological analyses), were collected yielding a total of 72 samples. Samples were collected in sterile 1000 ml polyethylene containers (those destined for biological analyses were fixed with 2 ml of 10 % formalin), and transported to the laboratory of Hydrobiology and Environment of the University of Yaoundé 1 and the Laboratory of Microbiology, Infectious diseases and Immunology of the Institute of Medical Research and Medicinal Plants Studies for physico-chemical and biological analyses respectively.

### 2.3 Physico-chemical analyses of water samples

Physicochemical variables were measured with the proper tools, adhering to methods, and suggestions by Rodier et al. (Rodier et al., 2009). The LAQUA Horiba P200 multi-parameter was used to monitor water temperature, pH, and electrical conductivity while dissolved oxygen was measured using an oximeter. Suspended Solids (MES), turbidity, ammoniacal nitrogen, nitrates, and orthophosphates were assessed in the lab using the HACH/DR 2010 spectrophotometer, while alkalinity, and oxidizability were determined using the volumetric approach (Rodier et al., 2009).

### 2.4 Parasite identification test

After 24 h of decantation, the resulting sediment was measured. Five (5) mll of each sediment was put into a 15 ml conical bottom tube and completed with distilled water till the limit followed by centrifugation at a speed of 2500 rpm for 5 min (Mbouombouo et al., 2019). With a pipette, 2 drops of lugol were added to the resultant sediment, homogenized and examined between slide and coverslip using 10× and 40× objectives under a light microscope for the presence of parasite-resistant forms (Mbouombouo et al., 2019). Parasite-resistant forms were identified following the WHO parasite identification key (World Health Organization, 1994).

### 2.5 Result expression

- Parasite prevalence (%) was calculated as proposed by (Hamit et al., 2008).
  $$P=\frac{\text{number of samples positive for}\;\mathrm{at}\;\text{least}\;\mathrm{one}\;\text{parasite resistant form}}{\text{total number of samples examined}}\;X\;100$$

Parasite density (eggs/L) was calculated following the formula proposed by Ajeagah et al. (Ajeagah et al., 2010)

x=yVx/Vy

where Vx = volume of sediment in 1 L sample, Vy = volume of observed sediment, y = number of eggs observed in Vy.

### 2.6 Statistical analysis

The correlation between helminths density and physico-chemical variables were evaluated using the Spearman correlation test. The Kruskal-Wallis test was used to compare mean values of physico-chemical parameters and parasite frequency. Principal Component Analysis (PCA) was applied to the physicochemical variables and to the composition of the population in order to group the sampling stations according to their biotic or abiotic similarities. These analyses were carried out with the R software version 4.0.5 and significance level was set at *p* ≤ 0.05.

## 3 Results

### 3.1 Global prevalence and relative densities of observed parasite resistant forms

**Fig. 2:**
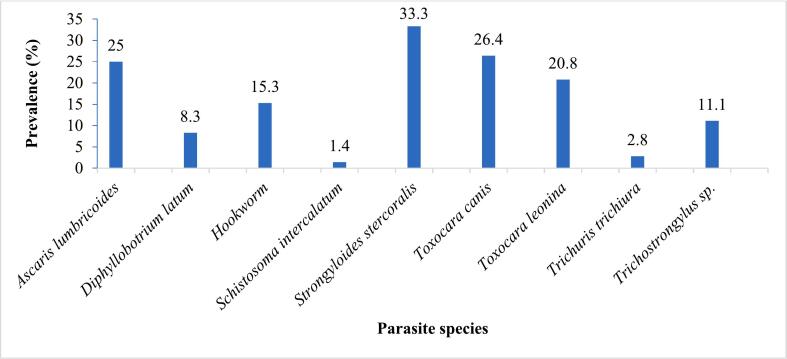
Global prevalence of observed parasite resistant forms.

**Fig. 3:**
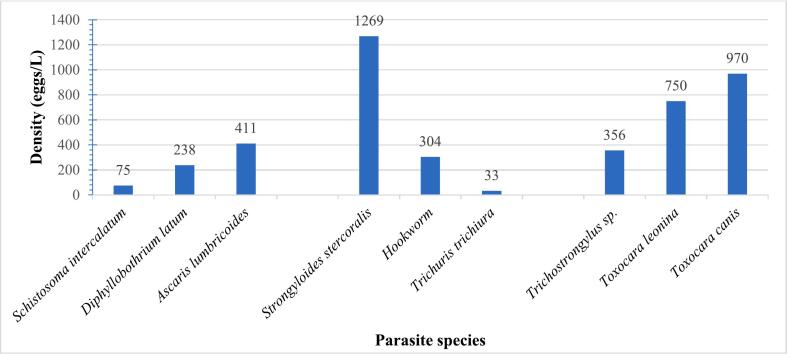
Relative densities of observed parasite resistant forms.

Globally, out of the 72 samples examined, 54 were positive for helminth parasite resistant forms accounting for a 75 % prevalence rate. In all, resistant forms of nine parasite species were recorded with the genus *Toxocara* being the most diversified with two species. *Strongyloides stercoralis* was the most prevalent (33.3 %) (Fig. 2) and most abundant (1269) species (Fig. 3) while *Schistosoma intercalatum* was the least prevalent (1.4 %) (Fig. 2) and the least abundant species was *Trichuris trichiura* (33) (Fig. 3).

### 3.2 Spatial variation of prevalence and average densities of parasite resistant forms

**Fig. 4:**
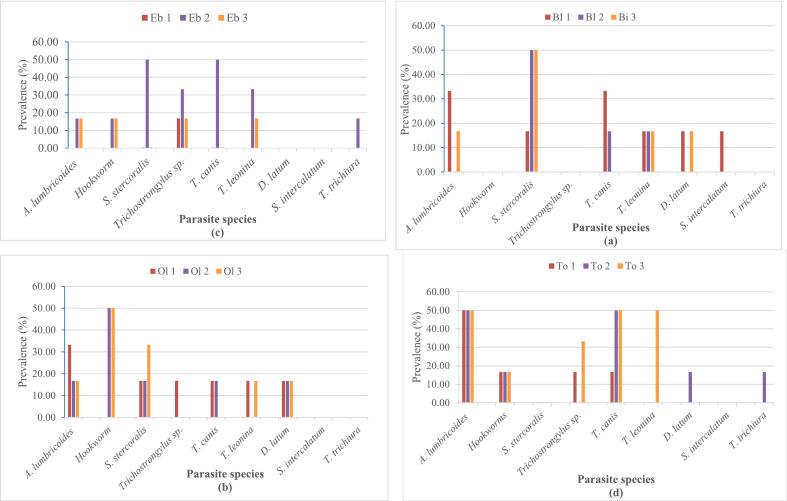
Spatial variation of prevalence of parasite resistant forms. (a = River Biyeme; b = River Olezoa; c = River Ebogo; d = River Tongolo)

**Fig. 5:**
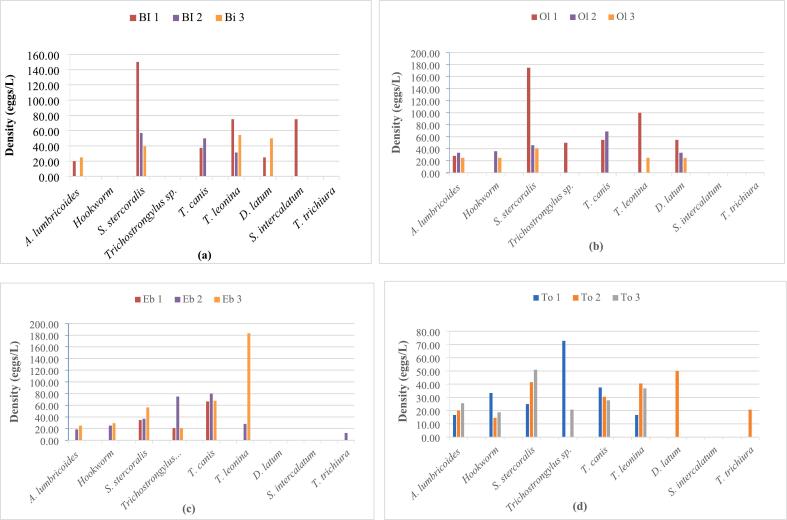
Spatial variation of average densities of parasite resistant forms. (a = River Biyeme; b = River Olezoa; c = River Ebogo; d = River Tongolo)

Spatially, the highest prevalence of 50 % was recorded for *A. lumbricoides* (at To 1,2,3), *T*. *leonina* (at To 3), *S. stercoralis* (at Bi 2,3, Eb 2), Hookworm (at Ol 2,3) and *T. canis* (at Eb 2, To 2,3) (Fig. 4). *Strongyloides stercoralis* was ever present with density fluctuating between 25eggs/L in To 1 and 175 eggs/L in Ol 1. *Toxocara leonina* presented the highest density of 183.33eggs/L at Eb 3 followed by *S. stercoralis* with 175 eggs/L and 150 eggs/L at Ol 1 and Bi 1 respectively (Fig. 5).

### 3.3 Temporal variation of prevalence and average densities of parasite resistant forms

**Fig. 6:**
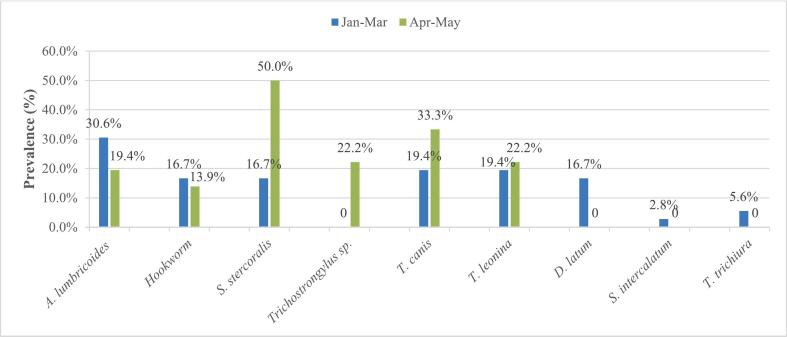
Seasonal variation of prevalence of parasite resistant forms. Jan = January, Mar = March, Apr = April, Jun = June.

**Fig. 7:**
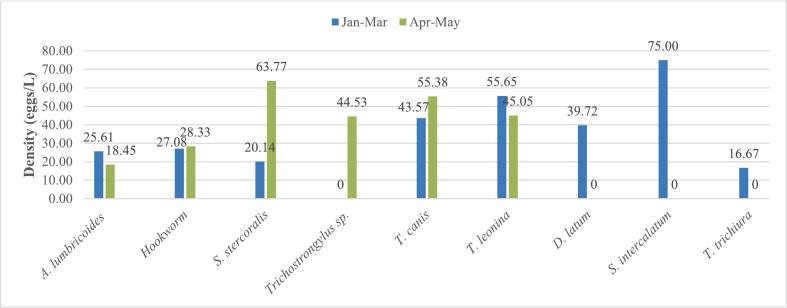
Seasonal variation of average densities of parasite resistant forms. Jan = January, Mar = March, Apr = April, Jun = June.

Seasonally, *Strongyloides stercoralis* presented the highest prevalence rate of 50 % followed by *T. canis* with 33.3 % both during the short rainy season (SRS), while *A. lumbricoides* had the highest prevalence rate of 30.6 % during the long dry season (LDS). Generally, prevalence did not vary significantly between both seasons. Highest mean densities for helminth eggs identified were those of *Schistosoma intercalatum* (75eggs/L) and *T. leonina* (55.65 ± 57.77eggs/L) during the long dry season compared to those of *S. stercoralis* (63.77 ± 44.73eggs/L) and *T. canis* (55.38 ± 47.65 eggs/L) during the short rainy season (Fig. 6). (See Fig. 7.)

### 3.4 Physico-chemistry of the rivers

The temperature of the waters ranged from 22.27 °C at station Ol 2 of river Olezoa in the short rainy season (April to June) to 28.10 °C in river Ebogo at stations Eb 3 in the long dry season (January to March). The Mann Whitney *U* test revealed a significant difference between both seasons (*p* = 0.019). Total suspended solids (MES) and turbidity varied from 9.33 to 52.67 mg/L and from 16.67 to 78.33 FTU with the highest values recorded in the rainy season (Table 2). The pH of the rivers fluctuated between 7.2CU during the LDS at station Ol 3 and 8.36CU during the SRS at station To 3. Globally, the rivers were fairly basic with an overall average of 7.72 ± 0.41CU. The Mann Whitney U test revealed no significant differences in electrical conductivity of the rivers between both seasons (*p* = 0.364) with a mean value of 398.23 ± 70.11 μS/cm. With regards to nitrates and orthophosphates, they ranged from 1.83 to 5.47 mg/L (3.11 ± 1.01 mg/L) and 62.27 to 2121.67 μg/L (385.57 ± 239.84 μg/L) respectively. Values of ammoniacal nitrogen in the rivers fluctuated between 1.88 mg/L (at To 1 in LDS) and 42.22 mg/L (at Ol 2 in SRS). Dissolved oxygen levels did not vary significantly between both seasons (*p* = 0.401) with percentages ranging from 2.63 to 38.28 (10.5 ± 6.8 %). Concerning alkalinity and oxydability, their highest values were recorded in the SRS. Their variation profile illustrated a range from 6.67 mg/L (Ol 2 in LDS) to 17.33 mg/L (Bi 2 in SRS) and from 1.18 mg/L (To 3 in LDS) to 16.64 mg/L (Ol 2 in SRS) respectively (Table 3).

**Table 2:** Variation of the physical variables of the rivers.

Rivers	Stations	Seasons	Temperature (°C)	Suspended solids (mg/L)	Turbidity (FTU)
Biyeme	Bi 1	LDS	23.30	25.00	49.67
	Bi 1	SRS	24.53	11.33	16.67
	Bi 2	LDS	26.50	27.00	47.33
	Bi 2	SRS	22.60	18.67	31.00
	Bi 3	LDS	24.20	28.00	34.33
	Bi 3	SRS	23.87	14.00	20.00
Olezoa	Ol 1	LDS	26.53	27.67	56.33
	Ol 1	SRS	24.73	52.67	78.33
	Ol 2	LDS	25.93	25.00	37.67
	Ol 2	SRS	22.27	14.33	28.67
	Ol 3	LDS	24.47	22.33	39.33
	Ol 3	SRS	24.47	21.67	34.33
Ebogo	Eb 1	LDS	27.50	18.00	28.33
	Eb 1	SRS	26.90	16.00	30.00
	Eb 2	LDS	27.67	9.33	22.33
	Eb 2	SRS	26.87	17.00	30.67
	Eb 3	LDS	28.10	11.67	26.67
	Eb 3	SRS	27.00	20.00	23.00
Tongolo	To 1	LDS	26.80	14.33	31.33
	To 1	SRS	25.53	18.00	30.00
	To 2	LDS	27.33	16.00	21.33
	To 2	SRS	23.93	34.33	45.33
	To 3	LDS	24.83	20.00	31.00
	To 3	SRS	25.33	13.33	21.00
***P* value**			**0.019**	**0.546**	**0.112**
**Accepted values for water quality parameters**			24	–	–

LDS = long dry season; SRS = Short rainy season; Bi1 = biyeme 1; Bi2 = biyeme 2; Bi3 = biyeme 3; Eb1 = Ebogo 1; Eb2 = Ebogo 2; Eb3 = Ebogo 3; Ol1 = Olezoa 1; Ol2 = Olezoa 2; Ol3 = Olezoa 3; To1 = Tongolo 1; To2 = Tongolo 2; To3 = Tongolo 3.

**Table 3:** Variation of the chemical variables of the rivers.

Rivers	Stations	Seasons	pH (CU)	Conductivity ((mg/L))	Nitrate (mg/L)	Phosphate μg/L	Ammoniacal nitrogen(mg/L)	Alkalinity (mg/L)	Dissolved O_2_ (%)	Oxidability (mg/L)
Biyeme	Bi1	LDS	7.34	480.20	2.90	116.60	6.79	10.67	13.40	3.36
	Bi1	SRS	7.93	474.87	2.63	197.00	7.07	15.33	15.97	5.02
	Bi2	LDS	7.37	493.20	3.53	213.67	9.59	14.00	4.67	3.89
	Bi2	SRS	8.01	472.03	3.63	2121.67	4.66	17.33	13.59	10.49
	Bi3	LDS	7.31	450.33	1.83	841.63	6.35	12.00	5.57	6.12
	Bi3	SRS	8.00	423.87	2.77	475.33	3.07	13.33	38.27	8.59
Olezoa	Ol1	LDS	7.21	607.37	4.77	171.60	8.78	14.00	2.63	3.36
	Ol1	SRS	8.23	497.97	5.43	247.00	5.12	16.67	4.50	11.64
	Ol2	LDS	7.29	298.13	2.33	107.80	6.37	6.67	4.67	2.17
	Ol2	SRS	8.07	234.50	2.00	445.33	42.22	9.33	9.27	16.64
	Ol3	LDS	7.20	393.93	4.00	194.70	5.69	9.33	3.33	4.08
	Ol3	SRS	7.99	317.25	7.10	512.67	3.37	12.00	5.53	9.24
Ebogo	Eb1	LDS	7.23	358.90	2.20	62.27	5.69	12.00	5.07	3.03
	Eb1	SRS	8.13	346.80	2.63	391.33	2.03	14.67	4.73	8.23
	Eb2	LDS	7.28	382.70	2.87	163.13	5.42	12.67	5.43	2.23
	Eb2	SRS	8.18	397.57	2.23	277.00	2.34	14.00	3.07	7.29
	Eb3	LDS	7.43	353.33	2.37	115.80	6.11	9.33	33.17	1.45
	Eb3	SRS	8.16	366.80	3.07	426.67	2.30	14.00	14.90	9.74
Tongolo	To1	LDS	7.31	213.80	2.53	195.73	1.88	7.33	4.83	1.65
	To1	SRS	8.21	273.80	2.03	403.67	2.02	12.00	14.30	9.33
	To2	LDS	7.31	427.50	2.27	190.93	4.61	12.67	21.77	2.44
	To2	SRS	8.28	411.27	5.47	480.33	3.18	14.67	5.13	12.76
	To3	LDS	7.34	433.20	1.97	635.17	4.98	14.00	7.43	1.18
	To3	SRS	8.36	448.30	2.13	266.67	4.20	16.67	10.77	9.84
**P value**			**<0.001**	**0.364**	**0.839**	**<0.001**	**<0.001**	**0.01**	**0.401**	**<0.001**
**Accepted values for water quality parameters**			6–9	–	10	0.1	0.1	–	90	–

LDS = long dry season; SRS = Short rainy season; Bi1 = biyeme 1; Bi2 = biyeme 2; Bi3 = biyeme 3; Eb1 = Ebogo 1; Eb2 = Ebogo 2; Eb3 = Ebogo 3; Ol1 = Olezoa 1; Ol2 = Olezoa 2; Ol3 = Olezoa 3; To1 = Tongolo 1; To2 = Tongolo 2; To3 = Tongolo 3.

### 3.5 Correlation between water physico-chemical properties, sampling stations and parasite resistant forms

The statistical tests conducted demonstrated and emphasized significant interdependencies between a few environmental factors and some parasite species found in the different rivers. Water pH, orthophosphate, organic matter and alkalinity revealed negative corelations with the occurrence of *Diphyllobotrium latum* (*r* = −0.375, −0.253, −462, −0.448 respectively). The occurrence of *Trichostrongylus* sp. were positively correlated with pH (*r* = 0.238) and negatively correlated with conductivity (*r* = −0.238) and ammoniacal nitrogen (*r* = −0.284). Hookworm eggs showed a negative correlation with both conductivity (r = −0.238) and alkalinity (*r* = −0.260). *A. lumbricoides* and *T. leonina* revealed a negative correlation with pH (*r* = −0.437) and orthophosphates (*r* = −0.336) while organic matter revealed positive correlations with the occurrence of *Strongyloides stercoralis* (*r* = 0.378) (Table 4).

**Table 4:** Correlation coefficients of Spearman between the evaluated water physico-chemical variables and parasite resistant forms.

	pH	Conductivity	Orthophosphate	Ammoniacal nitrogen	Alkalinity	Oxidability
*Ascaris lumbricoides*	**−0.437**^**⁎⁎**^	0.017	−0.149	−0.215	−0.138	−0.086
Hookworm	−0.151	**−0.238**^**⁎**^	−0.176	−0.146	**−0.260**^**⁎**^	0.045
*Strongyloides stercoralis*	0.062	−0.191	−0.022	−0.230	0.198	**0.378**^**⁎⁎**^
*Trichostrongylus* sp.	**0.238**^**⁎**^	**−0.238**^**⁎**^	0.021	**−0.284**^**⁎**^	0.067	0.194
*Toxocara canis*	−0.024	−0.056	−0.073	−0.173	0.104	0.032
*Toxocara leonina*	0.110	−0.027	**−0.336**^**⁎⁎**^	−0.001	0.058	−0.033
*Diphyllobotrium latum*	**−0.375**^**⁎⁎**^	0.150	**−0.253**^**⁎**^	0.083	**−0.462**^**⁎⁎**^	**−0.448**^**⁎⁎**^
*Schistosoma intercalatum*	0.011	0.169	−0.071	0.077	−0.203	−0.149
*Trichuris trichiura*	0.019	0.034	0.134	0.109	0.041	0.021

** = p (<0.001), * = p (<0.05), bold values = significant correlations.

**Fig. 8:**
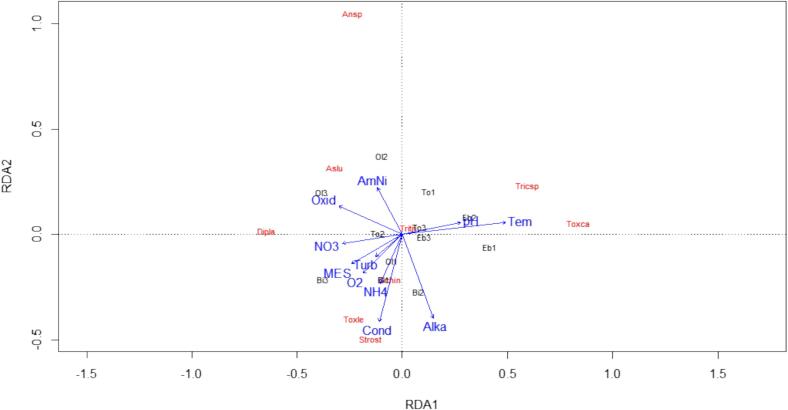
Correlation between water physico-chemical properties, sampling stations and parasite resistant forms. (Bi1 = biyeme 1; Bi2 = biyeme 2; Bi3 = biyeme 3; Eb1 = Ebogo 1; Eb2 = Ebogo 2; Eb3 = Ebogo 3; Ol1 = Olezoa 1; Ol2 = Olezoa 2; Ol3 = Olezoa 3; To1 = Tongolo 1; To2 = Tongolo 2; To3 = Tongolo 3; MES = total suspended solids; Tem = temperature; Alka = alkalinity; Cond = conductivity; AmNi = Ammoniacal nitrogen; O_2_ = dissolved oxygen; Tub = turbidity; NO_3_^−^ = nitrate; Oxid = oxidability; Aslu = *Ascaris lumbricoides*; Ansp = Hookworm; Strost = *Strongyloides stercoralis*; Tricsp = *Trichostrongylus* sp.; Toxca = *Toxocara canis*; Toxle = *Toxocara leonina*; Dipla = *Diphyllobotrium latum*; Schin = *Schistosoma intercalatum*; Tritri = *Trichuris trichiura)*

The redundancy analysis (RDA) shows the existence of two blocks for both abiotic and biotic variables. The two dimensions that characterize the parasite species and physicochemical parameters explain 61.77 % of the variables. Suspended solids, turbidity, nitrates, *Trichostrongylus* sp. and *Toxocara canis* were positively correlated to dimension 1 (37.32 %) and group the stations Eb 1, Eb 2 and Bi 3. Thus, an increase in concentration of the above parameters will enhance the growth and persistence of *Trichostrongylus* sp. and *Toxocara canis.* Aside from oxidability (soluble organic matter) which was negatively correlated, ammoniacal nitrogen, alkalinity, conductivity, Hookworm, *Strongyloides stercoralis*, and *Toxocara leonina* were positively correlated to dimension 2 (24.45 %). They characterize the stations Ol 2 and Bi 2. Hence, an increase in oxidability will have little or no effect on the growth of the parasites due to the fact that high concentrations of ammoniacal nitrogen, alkalinity and conductivity would have destroyed or rendered them inactive (Fig. 8).

## 4 Discussion

### 4.1 Biodiversity of parasite resistant forms

The present study reports that flood-prone areas in Yaounde are highly contaminated with helminth parasite resistant forms with 75 % global prevalence rate. They constitute two main phyla (Platyhelminths and Nemathelminths) grouped into eight families, eight genera and nine species. They include: *Strongyloides stercoralis*, *Toxocara canis*, *Schistosoma intercalatum, Ascaris lumbricoides*, *Toxocara leonina*, Hookworm, *Trichostrongylus* sp., *Diphyllobothrium latum*, and *Trichuris trichiura.* These results are similar to those obtained by Mbouombouo et al. (Mbouombouo et al., 2020) with a total of nine helminth species (*A. lumbricoïdes*, *Enterobius vermicularis*, *Ancylostoma* sp., *Strongyloides* sp., *Taenia* sp., *D. latum*, *Hyménolepis* sp., *Fasciola hepatica* and *S. haematobium*) registered in the Mufueh, Formuki, Mankon and Ayaba rivers of the North West Region of Cameroon. In general, this similarity could be due to several factors, including the sampling frequency, sampling methods and number of sampling stations. Numerous reasons, including the environment and the water facility's hygiene, could be responsible for the parasites' existence in the waters under study. Higher worm species will be activated by inadequate water and sanitation, which will be spread via contaminated soil and low personal hygiene (Alifia, 2021). According to Nurhayati et al. (Nurhayati et al., 2022) and Fahmi et al. (Fahmi et al., 2021), these parasites are believed to originate from faecal contamination of the waters caused by the use of latrines that contain faeces directly in contact with the ground or a septic tank at risk of leaking and resulting in water contamination in dug wells through soil seepage. Furthermore, the unavailability of toilets and the practice of open defecation in the rivers may result in the spread and growth of worm eggs in the running waters used for domestic purposes (Mbouombouo et al., 2019).

The dominance of Nematodes over the Trematode (*S. intercalatum*) is due to the high resistance of these species to environmental pressures. This can be explained by the presence of the three layers observed in most of these parasite resistant forms. The predominance of species of the Nematode class has been reported by Lalami et al. (Lalami et al., 2014), who have also pointed out that parasite eggs of this class are more dominant and resistant than those of Trematodes in water. The high densities of *S. stercoralis* larvae and *Toxocara eggs* obtained in the study could be justified by their ecology and biology, as well as by the high resistance of these parasites in the environment. The larvae of *Strongyloïdes* sp. can survive for many months in a humid environment and are highly adaptable to different milieus. The free parasitic phase of their lifecycle coupled with their double oesophageal bulge and ability to withstand heat, develop and reproduce optimally in damp milieus facilitate their multiplication thus a continuation of their lifecycle (Oyebamiji et al., 2018), particularly when conditions are favourable (temperature between 20 and 30 °C, neutral pH, average oxygenation and presence of organic matter). The high abundance of *Toxocara canis* and *Toxocara leonina* eggs observed during both seasons could be justified by the fact that stray animals such as cats and dogs roam freely in the city and contaminate the environment through their defecation (Otero et al., 2018). Also, the study by Ghomashlooyan et al. (Ghomashlooyan et al., 2015) revealed that temperatures between 15° and 30 °C favour the multiplication of *Toxocara* sp. eggs in the environment.

The high parasite densities obtained during the dry season can be attributed to water flow speeds, which decrease during this season, and to the accumulation of organic matter in the water. According to Mbouombouo et al. (Mbouombouo et al., 2019), parasite eggs are generally linked to organic matter suspended in the water. Thus, the decrease in parasite densities during the rainy season can be explained by the increase in water flow velocity, which causes particles to drift and, in turn, parasite eggs.

### 4.2 Influence of water physico-chemical parameters on parasite density

Our findings showed that the distribution of helminth eggs and larvae in these waters were linked to their physico-chemical characteristics. Temperature did not show a positive correlation with parasite densities although previous studies did indicate that temperatures between 15 and 25 °C will favour the development of parasites eggs and larvae. This shows that the development of parasite resistant forms depends on a variety of factors. Thus, the high density of *S. stercoralis* recorded in river Biyeme, Ebogo and Tongolo could be due to their high oxidability and suspended solids levels. This implies the waters are rich in nutrients needed by the pathogen for its survival and development. Therefore, the presence and persistence of viable pathogenic forms of this parasite will increase the probability of strongyloidiasis infection among those living in these sites. These results confirm those obtained by Ajeagah et al. (Ajeagah et al., 2010) indicating that there is a positive correlation between turbidity and the different pathogenic forms isolated from two streams subjected to organic pollution in Yaoundé. High densities of *Trichostrongylus* sp., eggs found in river Ebogo and Tongolo could be due to their neutral pH recorded. The World Health Organisation reports that parasites, in this case helminths, tolerate the pH range of 4.6 to 9.4CU and are still capable of hatching and developing into infectious stages (World Health Organisation, 2004). The increase in density of *T. canis* could be due to the increase in turbidity and alkalinity of the river. The negative correlations obtained between *D. latum* and water parameters such as electrical conductivity, pH, orthophosphate, alkalinity and oxidability would explain the low parasite loads observed in the stations. In fact, together with phosphorus, excess amounts of nitrates may accelerate eutrophication thus modifying the pH of the water environment (APHA, 1992). These mineral elements in the environment can increase the inactivation and destruction of resistant forms of parasites through their high capacity to penetrate their membranes (Mbouombouo et al., 2019). This is similar to results obtained by Letah et al. (Letah Nzouebet et al., 2016) however, it differs from that of Amandi & Uttah, (Amadi and Uttah, 2010) which revealed that conductivity had a positive link with resistant forms. This difference could be attributed to their different geographic locations. This implies that in such areas, the risks of these disease outbreaks will be negligeable even if the density of the parasite resistant forms is very high. The positive correlations obtained between suspended solids and the densities of certain parasites show that the presence of helminth eggs is generally linked to organic matter found in the waters (Ajeagah et al., 2016). It is therefore important to respect river water quality norms (Eaufrance, 2023) by monitoring river water quality parameters.

## 5 Conclusion

The results of this study show that the physico-chemical analyses of water were linked to the presence of helminth eggs and larvae, in the different rivers. This confirms that these waters are contaminated by faecal matter arising from domestic sewage (animals and humans) as well as piped latrines located on the river banks. This contamination also comes from runoff, which carries organic matter into the water, particularly during the rainy season. Hence, the increase in the organic content of the rivers is responsible for the abundance of parasite resistant forms. Therefore, a blend of sanitation and community health education are essential to effectively control human helminth infections particularly those of public health importance in our study area. It is thus in our best interest to stop littering the environment with human and other animal wastes, through the construction of faecal disposal facilities that are functional, accessible and appropriate for the local environment. We call on the government to be rigorous in following up the quality of wastewater liberated in the water environment.

## CRediT authorship contribution statement

**Noura Efietngab Atembeh:** Writing – original draft, Validation, Methodology, Investigation, Data curation. **Jean Patrick Molu:** Writing – review & editing, Visualization, Validation, Data curation. **Jeanne Valerie Emvoutou Maboulou:** Writing – review & editing, Visualization, Validation, Data curation. **Boris Fominyam:** Writing – review & editing, Visualization, Validation, Data curation. **Rodrigue Nanfack Dongmo:** Validation, Methodology, Data curation. **Gabriel Bertrand Zambo:** Writing – review & editing, Validation, Data curation. **Emilie Tchinda Tiecheu:** Writing – review & editing, Validation, Methodology, Investigation. **Jeannette Tombi:** Writing – review & editing, Visualization, Validation, Supervision. **Lucia Nkengazong:** Writing – review & editing, Writing – original draft, Visualization, Validation, Supervision, Methodology, Investigation, Funding acquisition, Conceptualization. **Serge Hubert Zebaze Togouet:** Writing – review & editing, Visualization, Validation, Supervision, Methodology, Conceptualization. **Jean Louis Essame Oyono:** Writing – review & editing, Validation, Supervision, Funding acquisition.

## Declaration of competing interest

The authors declare that they have no known competing financial interests or personal relationships that could have appeared to influence the work reported in this paper.
